# Advancing Real-Time Polyp Detection in Colonoscopy Imaging: An Anchor-Free Deep Learning Framework with Adaptive Multi-Scale Perception

**DOI:** 10.3390/s25247524

**Published:** 2025-12-11

**Authors:** Wanyu Qiu, Xiao Yang, Zirui Liu, Chen Qiu

**Affiliations:** 1 Hubei Key Laboratory of Digital Finance Innovation, Hubei Internet Finance Information Engineering Technology Research Center, School of Information Engineering, Hubei University of Economics, Wuhan 430205, China; 2 School of Computer of Science and Technology, Huazhong University of Science and Technology, Wuhan 430074, China; 3 School of Computer Science and Artificial Intelligence, Lanzhou University of Technology, Lanzhou 730050, China

**Keywords:** colonoscopy, polyp detection, anchor-free detection, real-time endoscopy

## Abstract

**Highlights:**

**What are the main findings?**

- A novel detector was developed that achieves state-of-the-art accuracy (98.8% mAP@0.5) and real-time speed (35.8 FPS) on a standard GTX 1080-Ti GPU, outperforming existing CNN and Transformer-based methods.
- The model’s performance is driven by three innovations: (1) a Cross-Stage Pyramid Pooling (CSPP) module for efficient multi-scale context aggregation, (2) a Weighted Bidirectional Feature Pyramid Network (WBFPN) for adaptive feature fusion, and (3) an anchor-free head with SDAIoU loss for precise localization without hand-tuned priors.

**What are the implications of the main finding?**

- The model’s high accuracy and real-time speed enable its practical use as a Computer-Aided Detection (CADe) tool in live colonoscopies, helping to reduce polyp miss rates and improve patient outcomes.
- This work provides an efficient and powerful CNN-based design paradigm that can match or exceed the performance of computationally expensive Transformer models in medical imaging applications.
- The anchor-free methodology is validated as a more robust and generalizable approach for detecting objects with high shape variance, simplifying model design and eliminating dataset-specific tuning.

**Abstract:**

Accurate and real-time detection of polyps in colonoscopy is a critical task for the early prevention of colorectal cancer. The primary difficulties include insufficient extraction of multi-scale contextual cues for polyps of different sizes, inefficient fusion of multi-level features, and a reliance on hand-crafted anchor priors that require extensive tuning and compromise generalization performance. Therefore, we introduce a one-stage anchor-free detector that achieves state-of-the-art accuracy whilst running in real-time on a GTX 1080-Ti GPU workstation. Specifically, to enrich contextual information across a wide spectrum, our Cross-Stage Pyramid Pooling module efficiently aggregates multi-scale contexts through cascaded pooling and cross-stage partial connections. Subsequently, to achieve a robust equilibrium between low-level spatial details and high-level semantics, our Weighted Bidirectional Feature Pyramid Network adaptively integrates features across all scales using learnable channel-wise weights. Furthermore, by reconceptualizing detection as a direct point-to-boundary regression task, our anchor-free head obviates the dependency on hand-tuned priors. This regression is supervised by a Scale-invariant Distance with Aspect-ratio IoU loss, substantially improving localization accuracy for polyps of diverse morphologies. Comprehensive experiments on a large dataset comprising 103,469 colonoscopy frames substantiate the superiority of our method, achieving 98.8% mAP@0.5 and 82.5% mAP@0.5:0.95 at 35.8 FPS. Our method outperforms widely used CNN-based models (e.g., EfficientDet, YOLO series) and recent Transformer-based competitors (e.g., Adamixer, HDETR), demonstrating its potential for clinical application.

## 1. Introduction

Colorectal cancer (CRC) represents a significant global health burden, ranking as the third most diagnosed malignancy and a leading cause of cancer-related mortality worldwide [1]. A crucial insight into CRC management is that most cases develop from benign adenomatous polyps through a well-documented adenoma-carcinoma sequence, a progression that can take several years. This presents a critical window for intervention. Colonoscopic screening, coupled with the timely removal of detected polyps, is the cornerstone of CRC prevention, proven to reduce its incidence by up to 88% and mortality significantly [2].

However, the effectiveness of colonoscopy is fundamentally limited by the endoscopist’s ability to visually identify all polyps. The colonic environment is visually complex; it is characterized by challenging lighting conditions, including specular reflections from the messy mucosa, motion blur from peristalsis or camera movement, and occlusions caused by haustral folds [3]. Under these demanding conditions, the polyp miss rate remains alarmingly high. Studies indicate that up to 26.3% of polyps are overlooked during standard procedures [4]. These missed lesions, particularly those that are small, flat, or located in less visible areas, represent a direct risk for interval cancers. Factors contributing to this challenge are multifaceted, including the subtle and varied morphology of polyps, suboptimal bowel preparation, and human factors such as operator fatigue and experience disparity. Consequently, the development of robust Computer-Aided Detection (CADe) systems powered by deep learning has emerged as a highly promising avenue to augment the endoscopist’s capabilities, enhance detection sensitivity, and ultimately improve patient outcomes [5].

In recent years, the field of automated polyp detection has been dominated by Convolutional Neural Networks (CNNs). To understand the positioning of our proposed framework, it is essential to trace the evolution of these detection paradigms and identify the specific clinical and technical gaps that remain unaddressed by the current techniques.

### 1.1. The Evolution from Anchors to Anchor-Free Designs

Early efforts in automated polyp detection leveraged general-purpose CNN-based object detectors, which can be broadly classified into two-stage and one-stage approaches. Two-stage methods, epitomized by Faster R-CNN [6] and R-CNN variants [7], first generate a sparse set of region proposals and then classify and refine each proposal [8]. While these methods established strong initial performance benchmarks, their sequential nature incurs significant computational latency, rendering them largely impractical for the real-time analysis required in live colonoscopy video streams. In response, one-stage detectors like the YOLO series [9,10] and RetinaNet [11] were introduced. These models forwent the explicit proposal stage to perform dense predictions directly on feature maps [12,13], offering efficiency more suitable for clinical deployment.

However, both these foundational architectures share a common, critical dependency: a set of pre-defined anchor boxes. While effective in natural image domains, this anchor-based paradigm introduces severe limitations in the specific context of polyp detection. Firstly, the dependence on hand-crafted hyper-parameters (scales and aspect ratios) is problematic. Polyps exhibit extreme morphological diversity, ranging from diminutive, flat lesions to large, pedunculated ones. This inherent variability necessitates tedious data-dependent calibration (e.g., k-means clustering) [14], which limits model generalizability across different datasets or populations. Secondly, and perhaps more critically, anchor-based methods struggle inherently with small object detection [15]. The mechanism relies on the Intersection-over-Union (IoU) overlap between anchors and ground truth. For small or flat polyps, a distinct mismatch in shape or a slight positional shift results in a drastically low IoU. Consequently, these objects are often assigned as negatives during training, exacerbating the intrinsic foreground-background imbalance [16]. This leads to a detector that is biased towards larger, easier-to-detect polyps while neglecting the clinically significant small lesions.

To overcome the rigidity of anchor-based designs, a new wave of anchor-free detectors has gained prominence. Innovative works like FCOS [17,18] and CornerNet [19], which regress bounding boxes from a center point or keypoints, demonstrated that high performance is achievable without anchor priors. This trend has been explored for polyp detection [20,21,22], successfully eliminating anchor-related hyper-parameters. Nevertheless, existing anchor-free polyp detectors often rely on standard regression losses (e.g., IoU loss) that treat all geometric errors equally. They lack mechanisms to explicitly model the geometric distortion of polyps or penalize aspect-ratio inconsistencies, which are critical when distinguishing flat polyps from normal mucosal folds.

To address these limitations, we propose a fully anchor-free detection head that directly predicts point-to-boundary distances, eliminating the need for anchor tuning. Crucially, to improve localization precision beyond standard anchor-free methods, we introduce a novel Scale-invariant Distance with Aspect-ratio IoU (SDAIoU) Loss. Unlike standard losses, SDAIoU provides robust geometric supervision that is invariant to polyp scale and highly sensitive to shape distortions, ensuring that even small and flat polyps are localized with high precision.

### 1.2. Addressing the Semantic Gap in Feature Fusion

Beyond the detection head, the ability to represent and see features at multiple scales is paramount. In deep CNNs, there exists a well-known “semantic gap” between shallow and deep layers. Shallow layers contain high-resolution spatial details (edges, textures) necessary for localization, while deep layers contain low-resolution semantic information (category presence) necessary for classification. Effective detection requires bridging this gap. Most detectors rely on the standard Feature Pyramid Network (FPN) [23] to merge these features. While FPN is a de-facto standard [22], its simple top-down pathway creates a unidirectional information flow. This is suboptimal for polyp detection because the visual cues for small polyps often reside entirely in lower layers, while the likelihood of existence resides in deeper layers; a single pathway inadequately merges these distinct signal types.

Advanced fusion architectures such as PANet [24] and BiFPN (EfficientDet) [25] introduced bottom-up pathways and weighted interactions. BiFPN, for instance, learns scalar weights to balance feature maps. However, we argue that simple scalar weights are insufficient for the medical domain. Colonic images are noisy, containing debris, water, and bubbles [3]. Scalar weighting applies the same importance to all channels in a feature map, effectively amplifying noise alongside signal in a “blind” manner. There is a need for a mechanism that can adaptively recalibrate features at a granular, channel-wise level to suppress noise before fusion [26].

To overcome the limitations of unidirectional and scalar-weighted fusion, we propose the Weighted Bidirectional FPN (WBFPN). This architecture integrates comprehensive bidirectional (shallow-deep and deep-shallow) pathways. More importantly, we upgrade the fusion nodes with a channel-wise attention block (Squeeze-and-Weight). This allows the network to not just weigh feature maps, but to selectively emphasize informative channels and suppress irrelevant background noise before fusion, providing a level of feature refinement absent in standard BiFPN.

### 1.3. Balancing Global Context, Inductive Bias, and Efficiency

Finally, precise differentiation of polyps from normal tissue requires long-range contextual information. Recently, Vision Transformers (ViTs) and DETR-like architectures [27] have emerged as a powerful alternative to CNNs. Models such as Adamixer [28], HDETR [29], and SQR-DETR [30] utilize global self-attention to model relationships across the entire image, achieving state-of-the-art accuracy.

However, the application of Transformers in clinical practice faces two major hurdles. First, Transformers lack the inherent “inductive biases” of CNNs, such as translation invariance and locality [31]. As a result, they are data-hungry and often struggle to generalize well on medical datasets, which are typically smaller than natural image datasets (e.g., COCO). Second, their performance comes at a prohibitive computational cost. The quadratic computational complexity and high memory requirements of self-attention mechanisms severely limit inference speed [32,33,34]. In clinical settings, where hardware is often constrained and latency is critical, achieving real-time performance (>30 FPS) is challenging with full Transformer architectures. There is a clear gap for a solution that captures the global context benefits of Transformers while retaining the data efficiency and inference speed benefits of CNNs. Existing context modules like ASPP [35] or SPP [36] offer a partial solution but are often computationally heavy or fixed in scale.

We aim to bridge the gap between Transformer-level context and CNN-level speed. We introduce the Cross-Stage Pyramid Pooling (CSPP) module. Instead of heavy self-attention or expensive atrous convolutions, CSPP integrates a fast, cascaded pooling structure within a cross-stage partial network [37]. This design efficiently harvests a rich pyramid of receptive fields to mimic global understanding with minimal computational overhead, maintaining the inductive bias of CNNs suitable for medical data.

### 1.4. Contributions and Paper Structure

Motivated by the discussion above, this paper presents a unified, real-time, anchor-free polyp detection framework. We argue that accurate and efficient polyp detection demands a system that is context-aware, carefully fused, and free from rigid anchor constraints. Evaluated on a large-scale clinical dataset, our model achieves superior performance (98.8% mAP@0.5, 82.5% mAP@0.5:0.95, and 98.3% mIoU) while operating at 35.8 frames per second (FPS) on a single GTX 1080 Ti workstation. The rationale described above leads to the following specific technical contributions:

- Cross-Stage Pyramid Pooling (CSPP): Addressing the high cost of Transformer-based context modeling, we design a lightweight module that captures a rich pyramid of receptive fields. By integrating cascaded pooling within a cross-stage partial network, CSPP enhances the deepest feature representation with negligible latency, making the model robust to scale variations without the overhead of self-attention.
- Weighted Bidirectional FPN (WBFPN): Addressing the semantic gap and the inadequacy of scalar-weighted fusion in noisy colonic environments, we propose an advanced fusion architecture. It integrates bidirectional pathways with a channel-wise attention mechanism, allowing the network to adaptively re-calibrate feature maps and balance low-level spatial details with high-level semantics more effectively.
- Anchor-Free Head with SDAIoU Loss: Addressing the limitations of rigid anchors and standard regression losses regarding small objects, we present a fully anchor-free regression paradigm supervised by a novel Scale-invariant Distance with Aspect-ratio IoU (SDAIoU) loss. This loss is specifically formulated to be invariant to polyp scale and sensitive to shape, ensuring robust detection of both flat and pedunculated polyps.

The remainder of this paper is structured as follows. Section 2 details the architecture and formal definitions of our proposed CSPP, WBFPN, and SDAIoU components. Section 3 presents the experimental setup, dataset description, ablation studies verifying the contribution of each module, and a thorough comparison with the state-of-the-art methods discussed above. Finally, Section 4 concludes the paper and discusses potential directions for future research.

## 2. Methods

### 2.1. Overall Architecture

**Figure 1:**
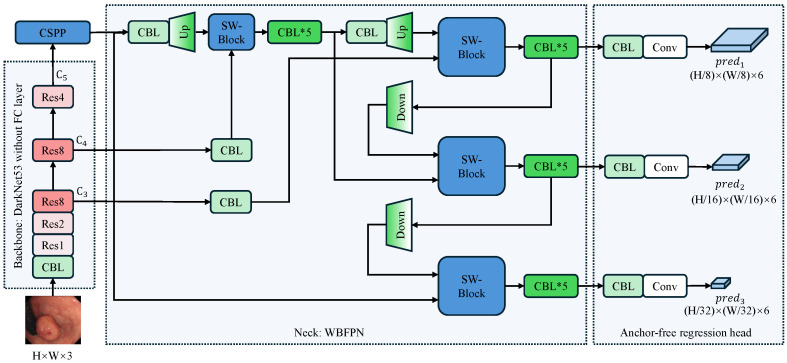
Overall architecture of the proposed anchor-free polyp detector. The arrows indicate the flow of feature maps. The input image undergoes initial processing via a Darknet-53 backbone that excludes fully connected layers, extracting multi-scale features $\left\{C_{3},C_{4},C_{5}\right\}$ with downsampling factors of 8, 16, and 32. The CSPP module enriches the top-level feature $C_{5}$ with multi-scale context, after which the WBFPN neck fuses all pyramid levels via bidirectional, channel-wise re-weighting. Finally, the anchor-free detection head directly predicts bounding boxes and confidence scores at three scales (${pred}_{1},{pred}_{2},{pred}_{3}$) without relying on pre-defined anchors. CBL denotes a Conv-BatchNorm-LeakyRELU block; SW-Block denotes the squeeze-and-weight block. Up and Down are used to resize feature maps through bilinear interpolation. The asterisk (*) indicates that the CBL module is stacked multiple times, and the number following “Res” signifies the repetition count of the residual block.

Figure 1 depicts the full pipeline of our proposed detector, which follows a backbone-neck-head design. The input RGB frame of size H×W×3 is first processed by a Darknet-53 backbone [9] that extracts multi-level features $\left\{C_{3},C_{4},C_{5}\right\}$ with downsampling factors of 8, 16, and 32, respectively. Subsequent components CSPP and WBFPN refine these features into a unified representation. Finally, an anchor-free detection head directly predicts a six-element vector at each spatial location of every $pred_{i}$ feature map. This vector comprises a confidence score, a label category prediction, and four directional distances $\left(\hat{L},\hat{T},\hat{R},\hat{B}\right)$ that define a bounding box relative to the center of the feature cell.

The selection of Darknet-53 as the backbone is predicated on a rigorous optimization of feature representation versus inference efficiency, a critical requirement for real-time colonoscopy. Unlike deeper architectures such as ResNet-101 [38], Darknet-53 achieves comparable detection accuracy with significantly reduced computational overhead, ensuring the rapid feedback necessary for clinical procedures. Furthermore, we prioritize a CNN architecture over Transformer-based alternatives like the Pyramid Vision Transformer [39] to leverage intrinsic inductive biases, specifically translation invariance and locality. These properties are vital for preventing overfitting on limited medical datasets, whereas Transformers’ self-attention mechanisms incur prohibitive latency costs. Instead, long-range context is efficiently managed by our CSPP module. Finally, in contrast to lightweight models like EfficientNet [40] that utilize depth-wise separable convolutions, Darknet-53 retains standard convolutions to preserve high representational capacity. This design is essential for capturing the fine-grained edge details and subtle textural variations required to distinguish flat polyps from the mucosal background, ultimately offering the superior balance of signal retention and computational throughput required for our anchor-free detector.

The network’s core logic follows a systematic flow: the backbone acts as a universal feature extractor; the neck, comprising our innovative CSPP and WBFPN modules, serves as the central hub for context aggregation and multi-scale feature refinement; and finally, the head performs anchor-free, per-pixel predictions. This decoupled design allows each component to be optimized for its specific task, collectively addressing the challenges of capturing diverse polyp morphologies in a computationally constrained environment.

It is worth noting that the $C_{5}$ feature map is uniquely processed by the CSPP module before entering the neck, while $C_{3}$ and $C_{4}$ are not. $C_{5}$ represents the highest-level semantic features with the largest receptive field but the lowest spatial resolution. In the complex colonic environment, differentiating polyps from folds requires distinguishing global context, which is best captured at this deep level. $C_{3}$ and $C_{4}$, conversely, are rich in spatial texture and boundary details. Our experiments indicated that applying the context-heavy CSPP to these shallower layers introduced computational redundancy without significant performance gains. Therefore, we apply CSPP only to $C_{5}$ to maximize semantic expansion, and then rely on the subsequent WBFPN to fuse this enhanced context with the spatial details of $C_{3}$ and $C_{4}$.

Throughout the network, particularly in the fusion neck and detection head, we utilize CBL (Conv-BatchNorm-LeakyReLU) blocks. These stacked blocks serve multiple critical functions: they perform channel dimensionality alignment for feature fusion, introduce non-linearity to decouple representations at different scales, and stabilize gradient flow during training.

### 2.2. Cross-Stage Pyramid Pooling (CSPP)

**Figure 2:**
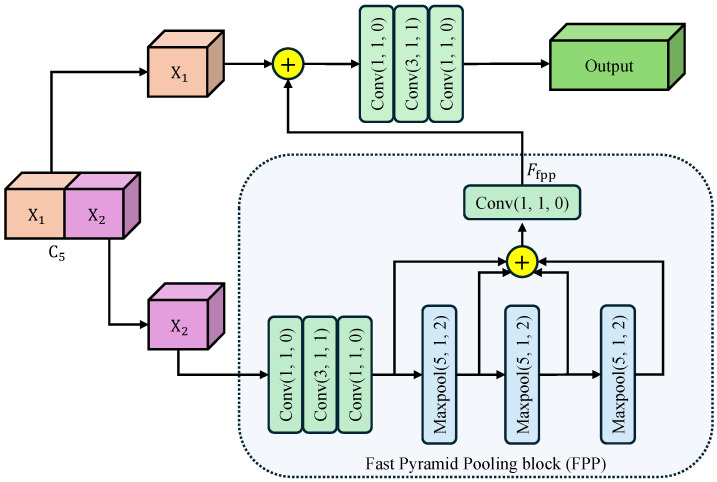
Architecture of Cross-Stage Pyramid Pooling (CSPP) module. The arrows indicate the flow of feature maps. Input feature is split into $X_{1}$ and $X_{2}$. $X_{2}$ undergoes cascaded max-pooling and convolutions to harvest multi-scale context, then re-fused with $X_{1}$ via a bottleneck to yield the enhanced output. Conv(k,s,p)/Maxpool(k,s,p) denotes a convolutional/maxpool layer with kernel size *k*, stride *s*, and padding *p*. The symbol ⊕ represents the concatenation of feature maps along the channel dimension.

To harvest rich multi-scale contextual information while keeping the computational budget low, we introduce CSPP as a plug-and-play component after $C_{5}$ feature map. As illustrated in Figure 2, CSPP is composed of three consecutive steps: feature splitting, fast pyramid pooling, and cross-stage re-fusion. Our motivation for designing CSPP stems from the limitations of conventional context modules like Spatial Pyramid Pooling (SPP) and Atrous Spatial Pyramid Pooling (ASPP). While effective, these modules often introduce significant computational overhead due to their parallel multi-branch structure. Furthermore, they are typically appended only to the deepest feature layer, creating a large semantic gap when these context-rich features are later fused with shallower, detail-oriented features. CSPP is engineered to mitigate these issues by embedding a pyramid pooling mechanism within a more efficient cross-stage partial network (CSP) architecture.

Feature Splitting: The input feature map $C_{5}\in R^{C\times H^{\prime }\times W^{\prime }}$ is first split along the channel dimension into two branches $X_{1},X_{2}\in R^{C/2\times H^{\prime }\times W^{\prime }}$. The expensive context aggregation operations are applied only to $X_{2}$, effectively halving the computational burden compared to standard global pooling. This reduces the parameter count and allows each branch to focus on complementary context patterns.

Fast Pyramid Pooling: The $X_{2}$ branch undergoes a Fast Pyramid Pooling (FPP) block consisting of three cascaded max-pooling layers. Each layer uses a kernel size k=5, stride s=1, and padding p=2, maintaining the spatial resolution of the feature map. This “SAME” padding strategy is crucial as it allows the pooled features to be concatenated directly with the original map. Furthermore, the cascaded structure allows us to approximate receptive fields of 5×5, 9×9, and 13×13 sequentially, without the cost of applying large independent kernels. Then, the three maxpooled features are concatenated with the original feature map along the channel dimension. Finally, a convolutional layer compresses the feature map channel to C/2, generating the output feature map $F_{\mathrm{fpp}}$.

Cross-Stage Re-fusion: The processed $X_{2}$ ($F_{\mathrm{fpp}}$) and the skipped $X_{1}$ are concatenated along the channel dimension, and the merged tensor is immediately fed into a bottleneck designed for cross-stage information re-calibration. The bottleneck consisting of determining convolutions: a 1×1 Conv for dimension reduction, a 3×3 Conv for spatial feature fusion, and a 1×1 Conv for channel expansion. This bottleneck ensures that the rich contexts from $X_{2}$ are seamlessly integrated with the preserved details of $X_{1}$. The split-and-re-fusion path not only preserves low-level structural details carried by $X_{1}$, but also injects the rich receptive-field pyramid aggregated in $F_{\mathrm{fpp}}$. Consequently, CSPP enhances the $C_{5}$ representation with negligible extra computational budget.

### 2.3. Weighted Bidirectional Feature Pyramid Network (WBFPN)

To fully exploit multi-level features while suppressing redundant information, we design WBFPN as the neck part of the detector. As presented in Figure 1, the WBFPN integrates two key components for efficient feature fusion. First, it utilizes bidirectional inter-scale connections to facilitate robust information exchange between feature maps of diverse resolutions. Second, the model incorporates learnable channel-wise weights, realized through an SW-Block, that adaptively adjust the contribution of each feature map. The design of WBFPN is motivated by the shortcomings of traditional feature fusion networks. The original FPN [23] utilizes a purely top-down pathway, which limits the flow of high-resolution spatial information to deeper layers. Path Aggregation Network (PANet) addresses this by adding a bottom-up path, but its fusion operations (e.g., simple addition) treat all incoming features as equally important. BiFPN [25] improves upon this with learnable scalar weights, but this single weight scales all feature channels uniformly. In polyp detection, where different channels may encode distinct visual cues (e.g., texture, color, vascular patterns), a more granular fusion mechanism is required. WBFPN provides this by introducing channel-wise attention at every fusion step, enabling a more intelligent and adaptive feature merging process.

Bidirectional Cross-Scale Fusion: Let $N=\{N_{3},N_{4},N_{5}\}$, in which $N_{3}=C_{3},N_{4}=C_{4}$ denote the feature hierarchy extracted from the backbone, and $N_{5}$ is the output of the CSPP module. WBFPN first augments the deep-to-shallow path of the original FPN with an additional shallow-to-deep pathway, yielding a fully connected directed acyclic graph. Formally, the intermediate feature $N_{\ell }^{\mathrm{td}}$ in the shallow-to-deep path is computed as

(1) $$N_{\ell }^{\mathrm{td}}=\mathrm{SW}\;\left(\mathrm{Resize}\left(N_{\ell +1}\right),N_{\ell }\right),$$

where Resize denotes bilinear interpolation upsampling or downsampling, and SW is the feature fusion block presented in next paragraph. The final fused feature $P_{\ell }^{\mathrm{out}}$ is given by

(2) $$N_{\ell }^{\mathrm{out}}=\mathrm{SW}\;\left(N_{\ell }^{\mathrm{td}},\mathrm{Resize}\left(N_{\ell -1}^{\mathrm{out}}\right)\right).$$

These repeated refinements allow low-level spatial details and high-level semantic cues to interact at every scale.

**Figure 3:**
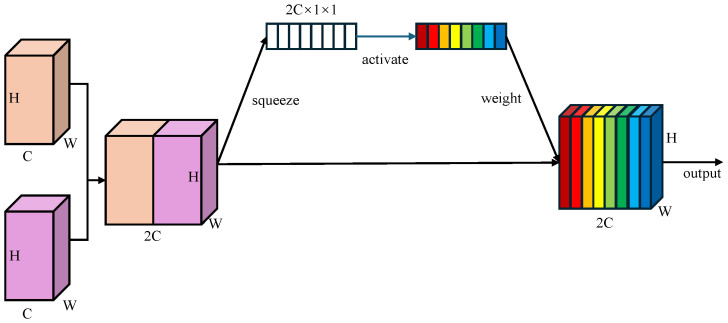
Architecture of squeeze-and-weight (SW) block. Global average pooling compresses spatial features into a channel descriptor, followed by a fully connected gating layer with RELU activation to re-weight channels, producing a channel-wise recalibrated feature map. Different colors represent different weights assigned to distinct feature map channels.

Learnable Channel-wise Weights: Unlike BiFPN which employs scalar attention, WBFPN attaches a lightweight squeeze-and-weight (SW) block to each feature fusion node. As shown in Figure 3, the SW-block first concatenate two feature maps along the channel dimension, resulting $N_{concat}\in R^{2C\times H\times W}$. Then, the global average pooling is used to squeeze the global spatial information $N_{concat}$ into a channel descriptor $s\in R^{2C}$. Next, a fully connected layer with RELU activation function produces the gating vector $w\in R^{2C}$, which is used to recalibrates the feature channels. The channel-wise re-weighting is performed as

(3) $${\tilde{N}}_{\mathrm{concat}}=w\circ N_{\mathrm{concat}},$$

where “∘” denotes the element-wise product broadcast along the spatial dimensions. By first concatenating the input features and then computing a joint channel-wise attention vector, the SW-block allows the gating mechanism to model inter-dependencies between the feature maps being fused. This is fundamentally more powerful than applying scalar weights or separate attention modules before fusion. It empowers the network to learn, for example, that when a certain semantic feature from a deep layer is present, specific spatial detail channels from a shallow layer should be amplified to refine polyp boundaries, while channels corresponding to noise or reflections should be suppressed. This dynamic, content-aware re-calibration is the core innovation of WBFPN over existing fusion networks. This channel-wise weighting mechanism enables the network to dynamically emphasize informative channels while suppressing less relevant ones during fusion.

### 2.4. Anchor-Free Regression Head

To remove the hand-crafted anchor priors and their associated hyper-parameter tuning, we propose a fully anchor-free regression head. The detection head receives feature maps of three scales from the WBFPN output, and then utilizes CBL blocks and a convolutional layer to directly output detection results ($pred_{1},pred_{2},pred_{3}$) on the feature maps with three different receptive fields. At each spatial location (x,y) on the output feature map ($pred_{1},pred_{2},pred_{3}$) is a vector of length 6, directly predicting confidence, category label and four coordinate values.

Point-to-Boundary Regression: The ground truth bounding box of the polyp, originally annotated on the input image, is projected to the coordinate system of each feature map $pred_{i}$. Mathematically, if the ground truth box on the input image is defined by coordinates $B_{gt}$, the corresponding projected box $Box_{i}$ on the *i*-th feature level is obtained by scaling down $B_{gt}$ by the stride factor $s_{i}$ (where $s_{i}\in \left\{8,16,32\right\}$). For each feature cell in the spatial dimension of any $pred_{i}$, its regression target is the distance from that feature cell to the left, upper, right, and bottom boundaries of $Box_{i}$.

**Figure 4:**
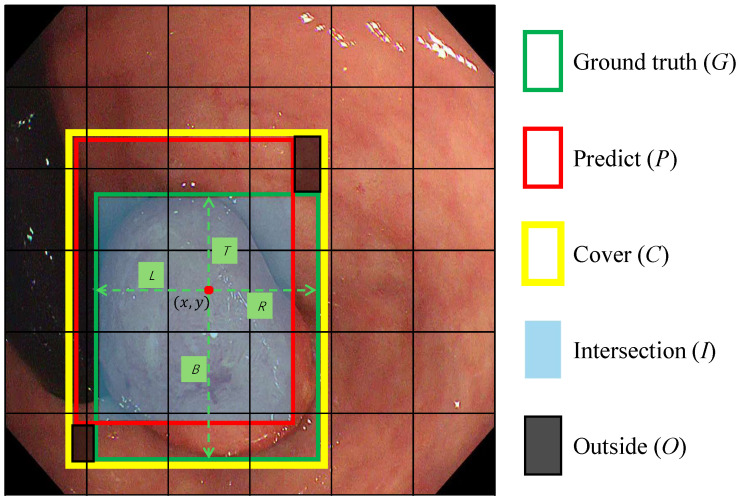
The scheme for calculating SDAIoU. *C* represents the smallest bounding box that covers both the Ground Truth (*G*) and the predicted box (*P*). *I* denotes the intersection of *G* and *P*. *O* denotes the region not covered by either bounding box. The red dot (x,y) represents the central coordinates of a feature grid cell in which the center of box *G* is located. The symbols L,T,R, and *B* denote the regression targets, which correspond to the distances from the red dot to the left, top, right, and bottom edges of box *G*, respectively.

Let (x,y) denote the spatial location of the feature grid cell where the center of a true polyp bounding box lies on the *i*-th feature map $pred_{i}\in R^{H_{i}\times W_{i}\times 6}$ with stride $s_{i}$. As depicted in Figure 4, instead of regressing offsets to pre-defined anchors, the proposed head predicts normalized distances to the four boundaries of the matched $Box_{i}$:

(4) $${\hat{D}}^{\left(i\right)}=\left\{{\hat{L}}^{\left(i\right)},{\hat{T}}^{\left(i\right)},{\hat{R}}^{\left(i\right)},{\hat{B}}^{\left(i\right)}\right\},$$

where

(5) $$\begin{array}{cc} {\hat{L}}^{\left(i\right)} & =\frac{x}{s_{i}}-x_{1}^{\left(i\right)},\;{\hat{T}}^{\left(i\right)}=\frac{y}{s_{i}}-y_{1}^{\left(i\right)},\\ {\hat{R}}^{\left(i\right)} & =x_{2}^{\left(i\right)}-\frac{x}{s_{i}},\;{\hat{B}}^{\left(i\right)}=y_{2}^{\left(i\right)}-\frac{y}{s_{i}}. \end{array}$$

Here $\left(x_{1}^{\left(i\right)},y_{1}^{\left(i\right)},x_{2}^{\left(i\right)},y_{2}^{\left(i\right)}\right)$ denote the top-left and bottom-right coordinates of $Box_{i}$, which is the ground-truth box mapped to the *i*-th level. $s_{i}=\left\{8,16,32\right\}$ are downsampling factors on three level feature maps.

This anchor-free formulation represents a paradigm shift from traditional detectors. It reframes bounding box detection as a more direct pixel-wise regression task, thereby eliminating the complex anchor matching strategies, IoU thresholding, and hyper-parameter tuning inherent to anchor-based methods. During training, we adopt a simple yet effective label assignment strategy: for a given ground-truth box, any spatial location (x,y) on a feature map that falls inside the projected area of the box is considered a positive sample for regression. To handle scale ambiguity, a ground-truth box is assigned to the single feature level $pred_{i}$ that best matches its scale range, preventing the model from learning conflicting targets for the same object.

SDAIoU Loss: Standard regression losses like L1 or L2 are suboptimal for bounding box regression as they treat the four box boundaries as independent variables and are sensitive to object scale. IoU-based losses provide a more holistic, scale-invariant metric. However, vanilla IoU loss stagnates when boxes do not overlap. Subsequent improvements like GIoU and DIoU introduced penalties for alignment and center point distance, but can still struggle with boxes of varying aspect ratios. To provide more robust supervision tailored for diverse polyp shapes, we propose a novel Scale-invariant Distance with Aspect-ratio IoU (SDAIoU) loss.

As depicted in Figure 4, our loss is composed of a custom geometric penalty $L_{\mathrm{geom}}$ and an explicit shape-consistency term $L_{\mathrm{shape}}$. First, we define a localization term that simultaneously rewards overlap and penalizes misalignment. The term (I−O)/C serves as a unified geometric score, where *I* is the intersection area, *O* is the area within the enclosing box *C* not covered by either the prediction or ground truth, and *C* is the area of the enclosing box. Maximizing this score intuitively encourages the predicted box to grow its overlap (*I*) while minimizing the “wasted space” (*O*) around it. Normalizing by *C* ensures this metric is scale-invariant. The localization loss $L_{\mathrm{geom}}$ is thus formulated as:

(6) $$L_{\mathrm{geom}}=1-\frac{I-O}{C}.$$

Second, to directly address the morphological diversity of polyps, we introduce an aspect-ratio consistency term. This component explicitly penalizes discrepancies in the shape of the predicted box, which is often neglected by IoU-based metrics alone. This is crucial because polyps can manifest as flat or elongated shapes, and standard losses may converge to a similar IoU with an incorrect aspect ratio. By enforcing aspect-ratio consistency, as visually supported by such constraints in Figure 4, we ensure the predicted box morphology aligns closely with the ground truth. The aspect-ratio consistency loss $L_{\mathrm{shape}}$ is defined as:

(7) $$L_{\mathrm{shape}}={\left(w-\hat{w}\right)}^{2}+{\left(h-\hat{h}\right)}^{2},$$

where $\left(\hat{w},\hat{h}\right)$ represent the predicted width and height of the bounding box, while (w,h) denote the ground truth width and height, respectively.

Finally, the complete SDAIoU loss combines these two components:

(8) $$L_{\mathrm{SDAIoU}}=L_{\mathrm{geom}}+L_{\mathrm{shape}}=1-\frac{I-O}{C}+{\left(w-\hat{w}\right)}^{2}+{\left(h-\hat{h}\right)}^{2}.$$

This composite loss function guides the model to learn not only the correct location and scale but also the specific shape of the polyp, resulting in more precise and morphologically faithful bounding box predictions, especially for non-square lesions.

Total Loss: The overall training objective is

(9) $$L=L_{\mathrm{cls}}+\lambda \;L_{\mathrm{SDAIoU}},$$

where $L_{\mathrm{cls}}$ is the loss for classification. The proposed polyp detector in this study focuses on locating polyps without classifying their categories. Therefore, $L_{\mathrm{cls}}$ represents the binary cross-entropy loss (BCEloss), which constrains the output categories for the logits produced by $pred_{i}$. The hyper-parameter λ serves as a crucial balancing factor between the localization task (supervised by $L_{\mathrm{SDAIoU}}$) and the classification task (supervised by $L_{\mathrm{cls}}$). Through empirical validation, we set λ=0.05 to ensure that the regression loss does not overpower the classification loss during training, leading to a stable convergence for both objectives.

## 3. Experiment

To rigorously validate the performance and efficiency of our proposed polyp detector, we conducted a series of comprehensive experiments. This section is organized as follows: first, we detail the experimental setup, including implementation specifics, training strategies, and the precise definition of our evaluation metrics. Second, we provide a more thorough description and analysis of the dataset used, highlighting its inherent challenges. Third, we present an in-depth analysis of our ablation studies to dissect the individual contribution of each proposed component, interpreting the quantitative results in the context of our design philosophy. Finally, we compare our model against a wide range of state-of-the-art CNN-based and Transformer-based detectors, offering a nuanced discussion of performance trade-offs that underscores the superiority of our approach for clinical applications.

### 3.1. Experimental Setup

All experiments were conducted on a workstation equipped with a Colorful GeForce GTX 1080 Ti GPU (11 GB VRAM; Colorful Technology, Shenzhen, China), an Intel Xeon E-2683 CPU (Intel Corporation, Santa Clara, CA, USA), and 64 GB of RAM. The workstation was procured from Wuhan, China. The operating system was Ubuntu 18.04 LTS. We employed Python 3.9.12 and PyTorch 1.13.1, accelerated by CUDA 11.6 and cuDNN 8.4. To ensure fairness and reproducibility, all models, including our own and the competing methods, were implemented and evaluated within the same standardized environment.

Training Details: The Darknet-53 backbone was initialized with weights pre-trained on the COCO dataset to accelerate convergence and improve generalization. Training proceeded for 300 epochs with a batch size of 16. We utilized the Stochastic Gradient Descent (SGD) optimizer with a momentum of 0.937 and a weight decay of $5\times 10^{-4}$. A learning rate schedule was employed, starting with a 3-epoch linear warm-up phase where the learning rate increased from 0.0 to an initial value of 0.01. Subsequently, a cosine annealing scheduler gradually decayed the learning rate to its minimum value over the remainder of the training, which has been shown to help the model settle into a more robust minimum. To mitigate overfitting and enhance the model’s robustness to variations in clinical settings, we applied a comprehensive suite of online data augmentation techniques, including random horizontal flipping, random scaling (within a range of 0.8 to 1.2), and color space adjustments (brightness, contrast, saturation).

Inference and Evaluation Metrics: During inference, all test images were resized to a fixed resolution of 640×640 pixels before being fed into the network. Initial raw predictions were filtered using a confidence threshold of 0.15. The remaining candidate boxes were then processed by Non-Maximum Suppression (NMS) with an Intersection over Union (IoU) threshold of 0.6 to eliminate redundant, overlapping detections for the same object. The inference speed, reported in Frames Per Second (FPS), was benchmarked on the test set with a batch size of 1 to simulate a real-world, sequential video processing scenario. Our evaluation relied on standard object detection metrics: Precision, Recall, mean Average Precision (mAP@0.5 and mAP@0.5:0.95), and mean IoU (mIoU). A detection was considered a True Positive (TP) if its IoU with a ground-truth box exceeded 0.5. mAP@0.5 provides a general measure of detection accuracy, while the stricter mAP@0.5:0.95 metric heavily penalizes localization inaccuracies, making it a key indicator of bounding box quality.

### 3.2. Dataset Description

The experiments were conducted on a comprehensive dataset that integrates both self-collected and public datasets to ensure a diverse and representative evaluation. Specifically, the dataset comprises two parts.

Union Dataset: This self-collected dataset contains 25 video sequences of clinical colonoscopy examinations. Initially, 25 sets of endoscopic data were annotated using Labelme software, yielding a total of 59,846 frames containing polyps. Through collaborative discussions with endoscopists, the bounding box annotations for the endoscopic data underwent multiple revisions, and inflammatory polyp images were meticulously excluded. Ultimately, 54,333 annotated frames were selected, characterized by high-quality annotations and minimal artifacts. Each frame is meticulously labeled with precise bounding box annotations of polyps.

SUN Dataset [41]: This public dataset, developed in collaboration with Nagoya University and Showa University Northern Yokohama Hospital, includes 100 video sequences of colonoscopy examinations with polyps, comprising 49,136 annotated frames.

To facilitate a thorough evaluation, the datasets were combined into a unified dataset of 103,469 frames. It was then partitioned into training, validation, and testing sets following a standard 8:1:1 split. Crucially, to prevent data leakage and ensure that the model is evaluated on entirely unseen patient cases, all frames from a single video sequence were assigned exclusively to one of the three sets. The dataset is characterized by a high degree of variability, mirroring real-world clinical challenges. It includes polyps of all major morphological types (e.g., pedunculated, sessile, flat) and a wide range of sizes, from diminutive lesions measuring only a few millimeters to large, conspicuous masses. The visual context is equally challenging, featuring variable illumination, specular reflections from the endoscope’s light source, the presence of liquid, stool, and air bubbles, as well as motion blur caused by endoscope movement and physiological motion.

### 3.3. Ablation Studies

Comprehensive ablation experiments were conducted to evaluate the individual and collective contribution of each proposed component: CSPP, WBFPN, and SDAIoU loss. All variants shared the same Darknet-53 backbone and were trained with identical hyper-parameters and data augmentation schemes for a controlled and fair comparison. Our baseline model utilizes the standard FPN [23] feature fusion structure and a conventional anchor-based detection head as seen in early YOLO models [42]. The progressive performance improvements are detailed in Table 1. Our analysis proceeds step-by-step.

Baseline Performance: The baseline model, without any of our proposed modules, achieved an mAP@0.5 of 89.2% with a Recall of 85.8% and a Precision of 87.4%. It operated at 38.4 FPS, serving as our performance and speed benchmark.

Effectiveness of CSPP: First, we integrated the Cross-Stage Pyramid Pooling (CSPP) module into the baseline. This addition yielded a substantial performance boost, with the mAP@0.5 surging by 6.5% to reach 95.7%. The improvement was driven by significant gains in both Recall (+4.6%) and Precision (+9.8%). This clearly demonstrates that enriching the feature representation with multi-scale contextual information is critical for distinguishing polyps from the complex background. This significant accuracy enhancement came at a negligible computational cost, with the FPS only marginally decreasing to 35.7.

Effectiveness of WBFPN: Next, upon the model with CSPP, we further incorporated our Weighted Bidirectional FPN (WBFPN). The introduction of this advanced neck architecture resulted in another clear performance increase. The mAP@0.5 rose by 1.1% to 96.8%, and Recall and Precision saw further improvements to 91.2% and 97.8%, respectively. This validates our hypothesis that a bidirectional fusion pathway with adaptive, channel-wise weighting enables a more effective synthesis of high-level semantic features and low-level spatial details, which is crucial for handling polyps of varying scales. The inference speed remained high at 34.5 FPS, confirming the efficiency of the WBFPN design.

Effectiveness of the Anchor-Free Head with SDAIoU Loss: Finally, we implemented our complete anchor-free design, where the head directly regresses the bounding box by predicting distances from a point to the four boundaries, supervised by our novel SDAIoU loss. This final step yielded the most significant performance leap, pushing the mAP@0.5 to an impressive 98.8%. The gain is fundamentally attributable to this anchor-free regression paradigm, which is more naturally suited to localizing objects with high variance in shape and scale, such as polyps. The SDAIoU loss is specifically engineered to provide robust supervision for this point-to-boundary regression task. Its scale-invariant and aspect-ratio-sensitive properties offer a much more effective optimization target than conventional losses, leading to superior localization accuracy. This is evidenced by the dramatic 7.6% surge in Recall to 98.8%, which indicates that the model, empowered by this design, becomes exceptionally proficient at detecting previously missed lesions. Remarkably, this advanced localization capability was achieved without any adverse impact on speed, with the final model operating at a real-time speed of 35.8 FPS.

In summary, the ablation study provides clear, empirical evidence that each component of Our method plays a distinct and vital role. The synergistic combination of CSPP for context, WBFPN for fusion, and an anchor-free head with SDAIoU for localization creates a highly effective and efficient detector specifically tailored to the challenges of polyp detection.

### 3.4. Comparison with Other Methods

As shown in Table 2, our proposed method establishes a new state-of-the-art across key metrics while sustaining real-time inference at 35.8 FPS. We analyze these results by comparing our model against two major categories of detectors.

Comparison with CNN-based Detectors: When compared against established CNN-based architectures, Our method demonstrates a significant leap in performance. It drastically outperforms canonical two-stage (Faster R-CNN) and one-stage (RetinaNet, YOLOv3) detectors across all accuracy metrics. For instance, it achieves a remarkable +17.9% gain in the strict mAP@0.5:0.95 metric over Faster R-CNN, highlighting its superior localization ability. More importantly, it also surpasses recent, highly optimized models like YOLOv9. While EfficientDet registers a higher FPS due to its lightweight backbone design, our model achieves a better overall clinical profile with higher recall, precision, and mAP scores. This indicates that our architectural choices (CSPP, WBFPN, SDAIoU) provide a more effective solution for the specific domain of polyp detection than the general-purpose scaling strategies employed by EfficientDet.

Comparison with Transformer-based Detectors: The comparison against Transformer-based methods is particularly insightful. These models, such as SQR-DETR and HDETR, leverage global self-attention and are known for their strong performance, as evidenced by their high mAP scores. Our method not only competes with but consistently matches or exceeds their accuracy. Specifically, we achieve the highest mAP@0.5 score (98.8%) and the best mAP@0.5:0.95 score (82.5%) in the entire comparison, demonstrating that a well-designed CNN can achieve a level of contextual understanding and localization precision that is on par with, or even superior to, state-of-the-art Transformers. The crucial differentiator, however, is computational efficiency. Our model operates at 35.8 FPS, which is substantially faster than all listed Transformer-based competitors. For example, it runs over 60% faster than SQR-DETR (21.4 FPS) and nearly twice as fast as HDETR (18.4 FPS). This performance gap is a direct consequence of the quadratic complexity of self-attention mechanisms, which makes them less suitable for real-time processing on standard clinical hardware. This result robustly validates our core motivation: it is possible to achieve top-tier accuracy without sacrificing the real-time efficiency that is non-negotiable for practical clinical deployment.

To further demonstrate the efficiency-accuracy trade-off design of our proposal, we additionally report the number of parameters (Params) and Giga Floating Point Operations (GFLOPs) for an input size of 3×416×416 in Table 2. As indicated, despite achieving higher accuracy than Transformer-based counterparts like SQR-DETR and HDETR, our model maintains significantly lower computational costs or offers a better speed/computation trade-off. This efficient architecture ensures that high-precision polyp detection is feasible even in resource-constrained clinical environments.

In conclusion, the comparative analysis confirms that our method occupies a unique and highly desirable position in the landscape of polyp detectors. It delivers the accuracy of sophisticated Transformer models while retaining the high efficiency of optimized CNNs, offering an ideal balance of performance and practicality for real-world colonoscopy applications.

## 4. Conclusions

This paper presented a novel anchor-free detector for real-time polyp detection in colonoscopy. The proposed framework integrates three key innovations: (1) the CSPP module that efficiently captures multi-scale contextual features through cross-stage partial connections and cascaded pooling, (2) the WBFPN architecture that adaptively fuses multi-level features via channel-wise attention, and (3) the anchor-free detection head optimized by SDAIoU loss for precise localization. Extensive evaluations on clinical datasets demonstrate state-of-the-art performance, with our method achieving 98.8% mAP@0.5 and 82.5% mAP@0.5:0.95 at 35.8 FPS on a single GTX1080Ti GPU workstation, outperforming both CNN-based and transformer-based competitors while maintaining real-time efficiency. This work bridges technical innovation with clinical requirements, ultimately enhancing early colorectal cancer prevention during routine colonoscopies. Despite the promising results, there are several avenues for future research.

Polyp Classification Subtyping: Extending the framework for histological classification of polyps (e.g., adenomatous vs. hyperplastic polyps) through multi-task classification heads. This requires curated datasets with pathological confirmation, to enable risk-stratified clinical decision support.

Temporal Video Analysis: Replacing frame-based processing with 3D convolutional networks or video transformers to exploit temporal context. Modeling inter-frame polyp dynamics and appearance consistency will improve detection robustness against transient artifacts and subtle lesions.

## Figures and Tables

**Table 1:** Ablation study on the test set. All models inherit the Darknet-53 backbone and are trained with identical hyper-parameters. The ✓/– mark indicates whether the corresponding module is integrated. **Bold** values indicate the best performance in each column.

CSPP	WBFPN	SDAIoU	Recall(%)	Precision(%)	mAP@0.5(%)	FPS
–	–	–	85.8	87.4	89.2	**38.4**
✓	–	–	90.4	97.2	95.7	35.7
✓	✓	–	91.2	97.8	96.8	34.5
✓	✓	✓	**98.8**	**98.2**	**98.8**	35.8

**Table 2:** Performance comparison on test set. The best score is in **bold**; the second best is underlined. “CNN” and “TF” denote CNN-based and Transformer-based detectors, respectively.

Model	Type	Params (M)	GFLOPs	Recall	Precision	mAP@0.5	mAP@0.5:0.95	mIoU	FPS
Faster R-CNN [6]	CNN	41.8	60	86.3	86.1	92.2	64.6	91.3	19.0
RetinaNet [11]	CNN	34.0	63	85.9	82.7	89.3	60.2	86.9	38.1
YOLOv3 [9]	CNN	61.6	66	85.5	87.0	91.0	58.8	89.8	38.9
YOLOv9 [43]	CNN	25.5	43	96.6	95.7	97.7	77.3	97.8	32.3
EfficientDet [25]	CNN	20.7	55	96.4	95.3	97.7	76.3	97.3	**44.1**
DETR [27]	TF	41.3	63	97.6	96.4	97.5	80.3	94.8	28.1
Adamixer [28]	TF	27.2	65	97.9	97.5	98.4	81.8	98.1	22.6
SQR-DETR [30]	TF	66.1	110.7	98.5	**98.2**	98.2	82.3	98.3	21.4
HDETR [29]	TF	42.6	70	98.2	98.0	98.3	82.3	**98.4**	18.4
ours	CNN	61.5	74.5	**98.8**	**98.2**	**98.8**	**82.5**	98.3	35.8

## Data Availability

The raw data supporting the conclusions of this article will be made available by the authors on request.
